# Employment of Tannin in Large Doses in Albuminous Anasarca

**Published:** 1860-01

**Authors:** P. Garnier


*Employment of Tannin in Large Doses in Albuminous Anasarca.* By Dr. P. Garnier.

Although the internal use of tannic acid is still very limited in France, its employment in large doses has been much recommended lately in other countries, and has been extended to numerous cases which, while proving its innoxious character, appear to exhibit it as possessing some totally new properties. It has been shown to be useful in all cases where it is required to arrest hemorrhages, to give tone to organism, or to remedy morbid secretions. It has been employed, for example with great benefit, in albuminuria, diabetes, and serous infiltrations.

From these considerations, Dr. Garnier has been induced to employ tannic acid in the albuminous anasarca consecutive to scarlatina; and he adduces several cases illustrative of this mode of treatment, drawn from his own experience and cases reported by other physicians. The cases all prove that in the general serous infiltration of the tissues complicated with albuminous urine there is a rapid and simultaneous disappearance of these two morbid phenomena under the influence of tannin alone, administered in a large dose. The conclusions drawn by Dr. Garnier are, that tannin, employed in doses of two to four grains a day ( ʒ ss. to ʒ j.) cures anasarca or œdema developed passively and occurring simultaneously with albuminous urine; that its curative action is manifested by abundant urine, gradually resuming its physiological characters, by perspiration, easy alvine evacuations, return of appetite, etc.; that these signs appear from the second day of the administration of the tannin; that, given in solution in doses of twenty to fifty centigrammes at a time, tannin causes no unfavorable symptoms affecting the digestive passages; and lastly, that the action of tannin appears to be exerted primarily upon the fluids of the economy, the albuminous principles of which it coagulates and renders plastic, and that its consecutive action on the solids appears to be tonic and astringent.—*Archieves Generales de Medicine, January,* 1859-

